# Effect of Auditory Constraints on Motor Performance Depends on Stage of Recovery Post-Stroke

**DOI:** 10.3389/fneur.2014.00106

**Published:** 2014-06-23

**Authors:** Viswanath Aluru, Ying Lu, Alan Leung, Joe Verghese, Preeti Raghavan

**Affiliations:** ^1^Department of Rehabilitation Medicine, New York University School of Medicine, New York, NY, USA; ^2^Center for the Promotion of Research Involving Innovative Statistical Methodology, Steinhardt School of Culture, Education and Human Development, New York University, New York, NY, USA; ^3^University of Pittsburgh Medical Center, Pittsburgh, PA, USA; ^4^Department of Neurology, Albert Einstein College of Medicine, Bronx, NY, USA; ^5^Department of Physical Therapy, Steinhardt School of Culture, Education and Human Development, New York University, New York, NY, USA

**Keywords:** bimanual movements, upper extremity, rehabilitation, motor learning/training, electromyography, task specificity, cerebrovascular disorders

## Abstract

In order to develop evidence-based rehabilitation protocols post-stroke, one must first reconcile the vast heterogeneity in the post-stroke population and develop protocols to facilitate motor learning in the various subgroups. The main purpose of this study is to show that auditory constraints interact with the stage of recovery post-stroke to influence motor learning. We characterized the stages of upper limb recovery using task-based kinematic measures in 20 subjects with chronic hemiparesis. We used a bimanual wrist extension task, performed with a custom-made wrist trainer, to facilitate learning of wrist extension in the paretic hand under four auditory conditions: (1) without auditory cueing; (2) to non-musical happy sounds; (3) to self-selected music; and (4) to a metronome beat set at a comfortable tempo. Two bimanual trials (15 s each) were followed by one unimanual trial with the paretic hand over six cycles under each condition. Clinical metrics, wrist and arm kinematics, and electromyographic activity were recorded. Hierarchical cluster analysis with the Mahalanobis metric based on baseline speed and extent of wrist movement stratified subjects into three distinct groups, which reflected their stage of recovery: spastic paresis, spastic co-contraction, and minimal paresis. In spastic paresis, the metronome beat increased wrist extension, but also increased muscle co-activation across the wrist. In contrast, in spastic co-contraction, no auditory stimulation increased wrist extension and reduced co-activation. In minimal paresis, wrist extension did not improve under any condition. The results suggest that auditory task constraints interact with stage of recovery during motor learning after stroke, perhaps due to recruitment of distinct neural substrates over the course of recovery. The findings advance our understanding of the mechanisms of progression of motor recovery and lay the foundation for personalized treatment algorithms post-stroke.

## Introduction

Stroke strikes one in six people worldwide. It is the leading cause of disability in the United States (1) and Europe (2), and the second leading cause of disability in the world (3). Hemiparesis is the most common reason for stroke-related disability, and the majority of individuals with hemiparesis have persistent deficits in hand function (4). There has been a recent surge in the availability of new rehabilitation strategies post-stroke. However, several large randomized controlled trials have failed to show the benefit of any one intervention over conventional treatment (5), and there remains a lack of understanding about how to select an appropriate treatment strategy for a given individual. While it is now accepted that task-specific training is an important aspect of a rehabilitation intervention, the constraints under which the task(s) should be practiced to be optimally therapeutic are not known. A constraint may be defined as the specific conditions under which a task is performed, for example, with one hand or both, with auditory/visual/multi-sensory feedback or without, etc. Task constraints are important because they regulate the information that is processed and assimilated by the nervous system, and the selection of constraints for any specific task may depend on the integrity and/or capacity to recruit specific neural substrates that facilitate processing of the relevant movement-related information. The stage of motor recovery, as measured by the level of motor impairment, may provide an indication for the type of task-specific constraints that are useful during practice for a given individual.

Fortunately, recovery of motor function after a hemiplegic stroke has been shown to follow a predictable pattern. Twitchell (6), Brunnstrom (7), and Fugl-Meyer et al. (8) described a hierarchical progression of recovery of patients who initially present with flaccid paralysis on one side of the body with areflexia. The reflex activity returns next and becomes heightened as spasticity emerges, and voluntary movements occur in stereotypical flexor and extensor synergy patterns. Spasticity then reaches its maximum level, producing characteristic patterns of stretch-sensitive responses such as spastic co-contraction. Eventually, the synergy patterns start to break up and spasticity begins to reduce as normal patterns of voluntary movement are restored. The emergence and disappearance of spasticity are thus important milestones in motor recovery (9, 10), although the severity of spasticity may vary considerably and temporary arrests in recovery or “plateaus” can occur at any stage (6).

Recent imaging studies further show how recovery processes unfold after a stroke [see Ref. (11) for review]. Early in recovery, the undamaged contralesional hemisphere shows increased activation (12–15), but eventually normal sensorimotor lateralization is restored in the stroke-affected hemisphere (16–18). Importantly, increases in neural activity in the contralesional motor areas in the first weeks after stroke correlate with better motor recovery in humans (19, 20) and monkeys (15), although persistent activation of the motor and non-motor areas in the contralesional hemisphere is noted in patients with poor motor outcome (18, 21). A recent longitudinal case study of a patient’s recovery over 21 months revealed continuous change in activation in the contralesional hemisphere with concomitant improvement in motor performance, whereas the ipsilesional hemisphere demonstrated significant change only toward the end of the study period (22). Taken together, these studies suggest that (1) redundant homologous pathways in the intact hemisphere can facilitate re-organization of the central nervous system, particularly in the earlier stages of recovery, and (2) that motor recovery occurs over a protracted and variable time period post-stroke. Hence the time since stroke may not reflect where an individual is in his or her recovery process.

Two kinds of bimanual training protocols have been developed to capitalize on contralesional cortical activity post-stroke. In active bimanual training, both arms move independently and simultaneously, requiring that individuals have at least some active movement on the paretic side. Active bimanual arm training combined with rhythmic auditory stimulation (BATRAC protocol) led to increased recruitment in the contralesional and ipsilesional hemispheres with concomitant improvement in performance of the paretic hand (23, 24). These data suggest that there may be a synergistic effect of bimanual and auditory constraints, but their individual contribution to performance improvement has not been ascertained. Rhythmic auditory stimulation by itself has also been found to be a useful adjunct to post-stroke rehabilitation (25–28). In active–passive bimanual training, the non-paretic arm drives movements of the paretic arm and leads to simultaneous mirror movements of both arms. Here, bimanual training occurred without auditory stimulation, was used to prime the ipsilesional motor cortex for subsequent training with the paretic arm, and also led to significant gains in arm function (29–32). An advantage of the active–passive approach is that it requires little active movement in the paretic arm and can therefore be used in individuals with significant paresis. Furthermore, the active–passive approach may be used to probe subsequent motor learning with the paretic arm. We have previously shown that motor learning is often impaired with the paretic hand, but may be temporarily restored after prior practice with the non-paretic hand (33).

In this study, we sought to determine the effect of various auditory constraints on bimanual-to-unimanual (paretic hand) learning in individuals at different stages of motor recovery post-stroke. Rhythmic stimulation with a metronome has been shown to improve spatiotemporal control of arm movements, perhaps via activation of brainstem–cerebellar networks (34, 35). However, several lines of evidence suggest that emotional drive via activation of limbic networks may also be an important predictor of motor performance (36) and post-stroke motor recovery (37). Music has been shown to activate a bilateral network of mesolimbic structures involved in processing emotions and reward information (38), and affective vocalizations have been shown to modulate attention via activation of pre-frontal–limbic networks (39). It is not clear when over the course of recovery one type of auditory stimulation versus another or no auditory stimulation will be beneficial. Hence, the objectives of this study were to: (1) characterize the stage of recovery in a disparate group of subjects with post-stroke hemiparesis using task-based kinematic measures, and (2) to examine how various types of auditory constraints interact with stage of recovery to facilitate learning with the paretic limb on a bimanual-to-unimanual learning task. Since voluntary wrist extension is frequently compromised post-stroke (40) and active wrist extension ability is predictive of hand function (41), we focused our task on training of wrist extension in the paretic hand. We hypothesized that auditory constraints that enhance emotional drive would facilitate learning of wrist extension with the paretic arm particularly in the early stages of recovery post-stroke.

## Materials and Methods

### Subjects

Twenty subjects with chronic post-stroke hemiparesis (at least 6 months prior to enrollment) were recruited through referrals from physicians at the Rusk Institute of Rehabilitation Medicine and through public advertisement. Subjects provided informed consent in accordance with the Institutional Review Board of the New York University School of Medicine. All subjects had at least 15° of passive and 5° of active wrist extension on the paretic side to perform unimanual movements, and they were screened to rule out hearing deficits prior to participation.

### Protocol

**Figure 1:**
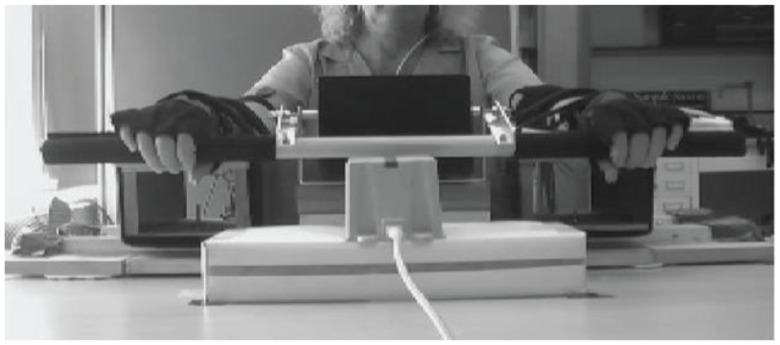
**Custom-made wrist extension trainer**.

**Figure 2:**
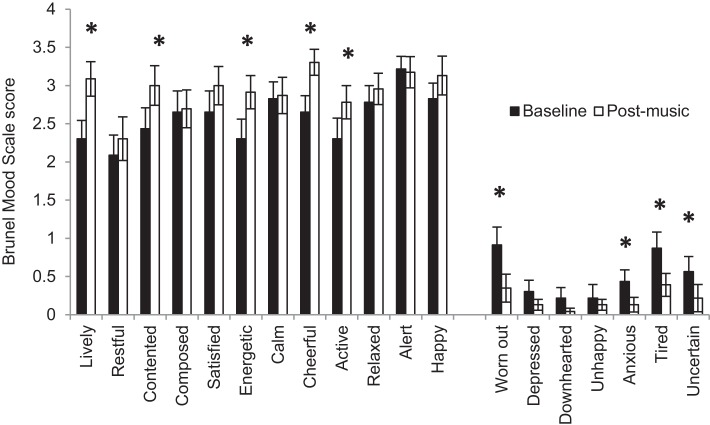
**Mean Brunel Mood Scale scores at baseline and after listening to self-selected music**. Error bars represent the standard error. *Represents statistically significant differences at *p* < 0.05.

The clinical assessments and experimental protocols were administered by well-trained research staff at the Motor Recovery Research Laboratory in the Rusk Institute of Rehabilitation Medicine. At the first visit, the Fugl-Meyer Scale (8) was used to assess upper extremity motor impairment; the Modified Ashworth Scale (42) assessed spasticity in the affected shoulder, elbow, wrist, and finger joints; active and passive range-of-motion at shoulder, elbow, wrist, and finger joints were measured using a goniometer (43), and the threshold for joint proprioception was also assessed. Depression and mood were assessed using the 15-item Geriatric Depression Scale, which has been recommended for the assessment of post-stroke depression in adults of all ages (44, 45), and the Brunel Mood Scale (BRUMS) (36), respectively. An appropriate tempo for the metronome beat was then determined by asking subjects to flex and extend their paretic wrist at a comfortable pace using a custom-made wrist trainer (Figure 1) for 15 s. Subjects then selected three familiar songs from public media to increase their feeling of vigor, happiness, and calmness. An up-tempo major key song that matched their metronome speed, or was in multiples of their metronome speed, was chosen to induce a positive mood-state. The BRUMS Scale was repeated after the subjects listened to their self-selected song to verify improvement in mood-states (Figure 2).

At the second visit, subjects performed repeated bimanual and unimanual (with the paretic hand) wrist flexion-extension movements using a custom-made wrist trainer. The device was designed to constrain movement of the wrist in the sagittal plane, and limit compensatory movement of the forearm and arm. The height of the chair was adjusted to keep the shoulders level and maintain proper alignment of the trunk. Table height and the position of the wrist trainer were maintained across all task conditions for each subject. Before the start of the experiment, subjects were informed that the goal of training was to facilitate wrist extension. Electromagnetic motion sensors (trakSTAR, Ascension Technology Corporation, Shelburne, VT, USA) affixed to the limb segments on each side measured wrist kinematics. Bipolar surface electrodes (DE 2.1, Delsys Inc., Natick, MA, USA) affixed over the flexor carpi ulnaris (FCU) and extensor carpi radialis longus (ECRL) muscles on each limb recorded electromyographic (EMG) signals. Video, kinematic, and EMG data were captured synchronously using The Motion Monitor (Innovative Sports Training Inc., Chicago, IL, USA), and analysis was performed offline using Spike 2 (Cambridge Electronic Design, Cambridge, England).

Wrist movements were performed under four different auditory conditions: (1) at baseline without auditory cueing; (2) to positively valenced affective “happy” sounds (baby’s laughter) recorded for 11 s and looped continuously, providing non-musical and non-rhythmic auditory stimulation; (3) to the self-selected up-tempo major key song chosen during visit 1; and (4) to a metronome beat set at each individual’s comfortable tempo. The subject was required to complete one cycle of wrist extension and flexion to each beat. Each condition consisted of 18 15-s trials of wrist flexion and extension, where subjects performed two bimanual trials followed by one unimanual trial with the paretic hand. A 20-s rest break was provided between each trial to prevent fatigue. The order of the conditions was counterbalanced across subjects, and subjects rated their fatigue levels after the completion of all trials for each condition.

### Data analysis

Kinematic data were sampled at 120 Hz and EMG data were sampled at 1206 Hz. The kinematic data were low pass filtered at 6 Hz and up-scaled to 1206 Hz using linear interpolation. The EMG data were filtered using a dual band pass filter (10–52.5 and 67.5–500 Hz) and the root mean square (RMS) of the signal was obtained for wrist flexion and extension phases of the movement separately. The EMG signals were normalized to the maximum amplitude recorded for each muscle across all trials and conditions (46, 47) to facilitate within- and between-subject comparisons. This method was chosen after extensive reliability testing of different methods of normalization (by Ying Lu). Movement speed, amplitude of wrist extension, wrist extensor activation (RMS of agonist, ECRL), wrist flexor activation (RMS of antagonist, FCU) during extension, and co-activation (defined as RMS of antagonist, FCU/RMS of agonist, ECRL) were the variables used in the analyses. Recognizing that the subjects may present at various stages of recovery at the time of the study, we used hierarchical cluster analysis with the Mahalanobis metric (48) based on baseline wrist kinematics to stratify subjects into groups. The stratification scheme corresponded well with recovery characterized by the Fugl-Meyer Scale as shown in the results below. We then fit linear mixed effect models with group interactions and individual random effects to assess: (1) differences among subject clusters, and (2) learning rates across repeated unimanual trials with the paretic hand after bimanual priming with the four auditory conditions. Learning rate on unimanual trials was defined as the slope of the linear trend fit over the six unimanual trials. All the statistical analyses were conducted using R (v. 2.15.1). The R package “lme 4” was used for the mixed effect model estimation. To control for multiple comparisons but preserve statistical power (due to low sample size in the subgroups), we present all results but choose to interpret results with marginal statistical significance (0.01 < *p* < 0.05) with caution.

## Results

**Figure 3:**
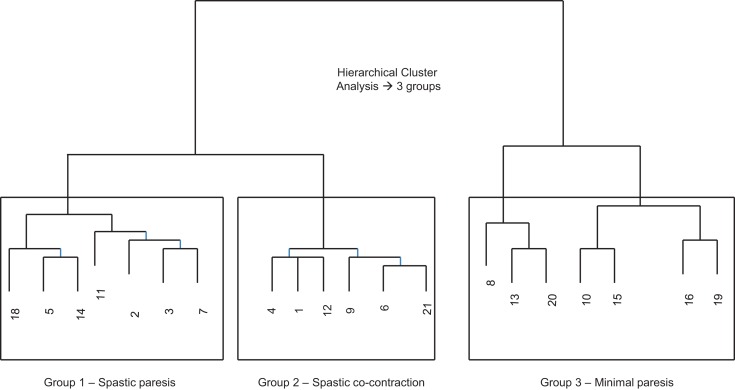
**Cluster dendrogram from hierarchical cluster analysis using the Mahalanobis metric based on speed and amplitude of wrist extension on the first trial with the paretic hand at baseline (without auditory stimulation)**. Three distinct groups emerged.

Our first objective was to characterize the stages of recovery across a disparate group of patients with post-stroke hemiparesis. Since wrist kinematics provide direct, objective, and reliable measures of movement ability in the paretic hand, we used the movement speed and extent of wrist extension from the first trial with the paretic hand under the baseline condition (no auditory cueing) to perform hierarchical cluster analysis (48), which stratified subjects into three distinct groups (Figure 3).

Clinical metrics (Table 1) showed clear differences across the three groups. The Fugl-Meyer scores were lowest in group 1, followed by group 2, and then group 3 (*p* = 0.047). Active wrist motion, measured using goniometry separately from the wrist extension task, showed that both wrist flexion and extension were surprisingly lowest in group 2, intermediate in group 1, and highest in group 3 (*p* < 0.001). Spasticity at the wrist flexors was, however, highest in group 1, and similar in groups 2 and 3 (*p* = 0.066). There were no significant differences among the three groups in joint proprioception at the wrist, depression scores, mood scores, tempo of the metronome beat or selected song, or fatigue levels. Note that the mean time since stroke was also not different across the three groups (*p* = 0.89).

**Table 1:** **Clinical characteristics of subjects: ^a^Sub, subjects in each group (see also Figure 2); ^b^age, in years; ^c^H/H, handedness/hemiparesis; ^d^TSS, time since stroke in months; ^e^FMS, Fugl-Meyer score, values represent total upper extremity scores out of a maximum of 66/hand and wrist score out of a maximum of 30; ^f^AROM, active range-of-motion in degrees at the wrist measured with a goniometer; ^g^MAS, modified Ashworth Scale measured at the wrist. Lesion location and stroke subtype obtained from: ^h^radiology reports and ^i^medical history narrative from subject**.

		Sub^a^	Age/sex^b^	H/H^c^	Stroke location/subtype	TSS^d^	**FMS^e^**	**AROM^f^**	**AROM**	**MAS^g^**	MAS
								Flexion	Extension	Flexors	Extensors
Group 1	Spastic paresis	2	43/F	R/R	L frontal hge^h^	8	**21/8**	**20**	**15**	**3**	1
		3	43/M	L/R	L subcortical hge^i^	N/A	**35/20**	**20**	**15**	**3**	1
		5	62/F	R/L	R parietal hge^h^	71	**48/22**	**15**	**15**	**3**	2
		7	36/M	R/R	L MCA infarcts with hge^h^	37	**38/20**	**20**	**20**	**1**	1
		11	65/M	R/R	L BG infarct^h^	50	**51/21**	**33**	**35**	**2**	1
		14	52/M	R/R	L IC occlusion^i^	123	**28/10**	**20**	**15**	**3**	1+
		18	60/F	R/L	R cerebral hge^i^	84	**33/18**	**20**	**10**	**2**	2

		Mean	51.6			62.2	**36.3/17**	**21.1**	**17.9**	**2.4**	1.4
		(SD)	(11.2)			(39.9)	**(10.6/5.6)**	**(5.6)**	**(8.1)**	**(0.8)**	(0.5)

Group 2	Spastic co-contraction	1	28/F	R/R	L MCA infarct^h^	44	**51/21**	**10**	**5**	**1**	0
		4	46/F	R/L	R MCA hge^i^	77	**42/20**	**20**	**10**	**2**	2
		6	61/M	R/R	L cerebral hge^i^	18	**37/21**	**15**	**15**	**3**	3
		9	54/M	R/R	L lacunar infarct^h^	49	**54/27**	**20**	**10**	**1**	1
		12	56/M	R/R	L lacunar infarct^h^	51	**62/27**	**10**	**5**	**1+**	1+
		21	87/F	R/L	R MCA infarct^h^	84	**25/5**	**15**	**5**	**1**	2

		Mean	55.3			53.8	**45.2/20.1**	**15.0**	**8.3**	**1.6**	1.6
		(SD)	(19.3)			(23.9)	**(13.3/8.0)**	**(4.5)**	**(4.1)**	**(0.8)**	(1.0)

Group 3	Minimal paresis	8	47/M	R/R	L IC occlusion^i^	71	**35/25**	**30**	**25**	**3**	1
		10	69/F	R/R	L thalamic infarct^h^	24	**58/25**	**50**	**55**	**1**	1
		13	42/F	R/L	R IC occlusion^h^	30	**40/20**	**50**	**70**	**2**	2
		15	71/M	R/L	R MCA infarct^h^	37	**55/20**	**50**	**45**	**1**	1
		16	41/M	R/R	L BG hge^h^	192	**65/29**	**60**	**50**	**1**	1
		19	59/M	R/R	L thalamic infarct^h^	36	**59/26**	**75**	**60**	**1**	1
		20	62/M	R/R	L MCA infarct^h^	69	**60/27**	**40**	**30**	**1**	1.1

		Mean	55.9			65.6	**53.1/24.5**	**50.7**	**47.9**	**1.4**	1.1
		(SD)	(12.5)			(58.8)	**(11.2/3.4)**	**(14.3)**	**(16.0)**	**(0.8)**	(0.4)

*P*-value across the three groups						0.89	**0.047**	**<0.001**	**<0.001**	**0.066**	0.502

*Bolded variables showed statistically and/or clinically important differences across the three groups*.

**Figure 4:**
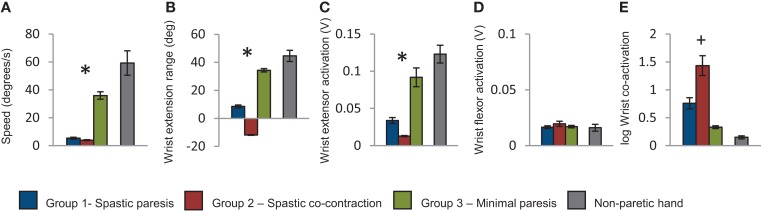
**Group means computed for the first trial with the paretic hand**. **(A)** Speed of wrist extension in degrees per second; **(B)** extent of wrist extension in degrees; **(C)** root mean square of wrist extensor muscle activation during wrist extension; **(D)** root mean square of wrist flexor muscle activation during wrist extension; **(E)** log of wrist co-activation computed as ratio of wrist flexor to extensor muscle activation. The blue bars represent the group with spastic paresis, which had the lowest Fugl-Meyer scores, the red bars represent the spastic co-contraction group with intermediate Fugl-Meyer scores, and the green bars represent the minimal paresis group, which had the highest Fugl-Meyer scores. Values for the non-paretic hand are shown in gray for reference. Error bars represent the standard error. *Represents differences between the three groups at *p* < 0.05, and ^+^represents differences between the three groups at *p* < 0.1.

Baseline performance metrics on the wrist extension task also showed clear differences across the three groups. Movement speed was higher in group 3 compared to groups 1 and 2 (*p* < 0.001, Figure 4A). Extent of wrist extension was lowest in group 2 (where attempted wrist extension produced paradoxical flexion), intermediate in group 1, and highest in group 3 (*p* < 0.001, Figure 4B). Wrist extensor muscle (ECRL) activation was also lowest in group 2, intermediate in group 1, and highest in group 3 (*p* = 0.047, Figure 4C), whereas wrist flexor muscle (FCU) activation was not differentiated in the three groups (*p* = 0.877, Figure 4D). Co-activation between wrist extensor and flexor muscles was highest in group 2, intermediate in group 1, and lowest in group 3 (*p* = 0.07, Figure 4E). Taken together, the baseline performance and clinical metrics enabled characterization of recovery patterns into the three descriptive groups below.

### Group 1 – spastic paresis

In this group, performance on the paretic side (Figure 4, shown in blue) relative to the non-paretic side showed low movement speed (~10%), moderate wrist extension (~20%), moderate activation in the wrist extensor (~30%), and five times greater co-activation. Clinically, these subjects had the lowest Fugl-Meyer scores (range 21–51), but had 15–33° of active wrist flexion and 10–35° of active wrist extension. Spasticity was observed predominantly in the wrist flexors. Lesion location and stroke subtype (Table 1) suggest that these subjects had very severe strokes that were caused predominantly by intracerebral hemorrhage (subject #s 2, 3, 5, 18) or hemorrhagic transformation of ischemic infarcts (subject # 7).

### Group 2 – spastic co-contraction

In this group, performance on the paretic side (Figure 4, shown in red) relative to the non-paretic side showed very slow movement speed (~7%), paradoxical wrist flexion on attempted wrist extension (−27%), minimal activation of the wrist extensor muscle (~10%), and ~10 times greater co-activation. Clinically, these subjects had higher Fugl-Meyer scores (range 25–62) than those in group 1. However, they had only 10–20° of active wrist flexion and 5–15 of active wrist extension. Spasticity was distributed equally in both wrist flexors and extensors for the most part. Lesion location and stroke subtype (Table 1) suggest that these subjects had moderately severe strokes caused predominantly by infarcts in the MCA territory (subject #s 1, 9, 12, 21).

### Group 3 – minimal paresis

In this group, performance on the paretic side (Figure 4, shown in green) relative to the non-paretic side showed relatively high movement speed (~60%), substantial wrist extension (~77%), and wrist extensor activation (~75%), and twice the co-activation as the non-paretic side. Clinically, these subjects had the highest Fugl-Meyer scores (range 35–65) and the greatest range of active wrist flexion (30–75°) and extension (25–70°) of the three groups. Lesion location and stroke subtype (Table 1) suggest that these subjects had a mixed variety of strokes predominantly in the MCA territory.

**Figure 5:**
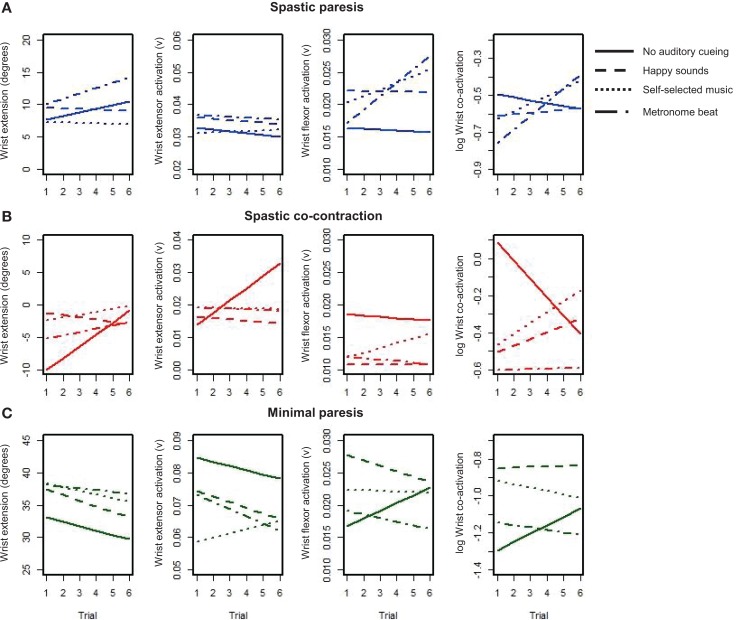
**Trendlines of wrist extension performance variables over six repeated trials with the paretic hand under each condition for the three groups: (A) spastic paresis (blue); (B) spastic co-contraction (red); (C) minimal paresis (green)**. The four conditions are represented by the different patterned lines.

Our second objective was to examine how different types of auditory stimuli interact with bimanual training to facilitate subsequent learning with the paretic limb in the three groups. Subjects performed six cycles of two bimanual trials followed by one unimanual trial with the paretic hand, where each trial consisted of multiple repeats of wrist flexion-extension over 15 s. We were interested in the changes in wrist extension, wrist extensor activation, wrist flexor activation, and co-activation over the six unimanual trials for each of the auditory conditions (represented by the different line patterns, see Figure 5). The mean level of the trend lines provides an indication of the amplitude of overall performance, whereas the slope of the trend lines quantifies the rate of learning on the paretic side. A positive slope suggests sustained improvement whereas a negative slope suggests reduced performance under that constraint. Subjects in the spastic paresis (Figure 5A) and spastic co-contraction (Figure 5B) groups started with low or negative wrist extension, but showed sustained improvements under certain auditory constraints. Subjects in the minimal paresis group (Figure 5C), showed good wrist extension at first, but did not improve much over the repeated trials.

**Figure 6:**
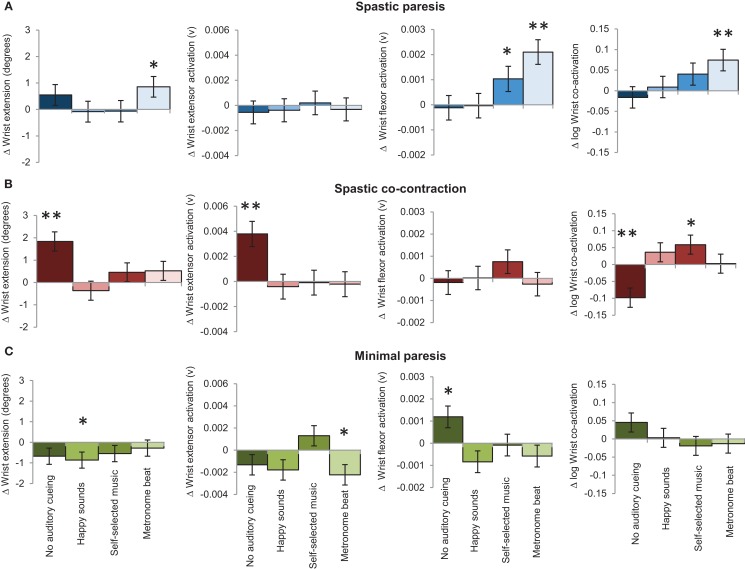
**The bars represent the mean slopes showing the effect of auditory stimulation on bimanual-to-unimanual learning for wrist extension performance variables in the three groups: (A) spastic paresis (blue); (B) spastic co-contraction (red); (C) minimal paresis (green)**. Error bars represent the standard error. **Represents differences between the three groups at *p* < 0.01, and *represents differences between the three groups at *p* < 0.05.

The slope (unit change per trial) succinctly summarizes which auditory conditions couple with bimanual training for sustained improvement on the paretic side in the three groups (Figure 6). In the spastic paresis group (Figure 6A), wrist extension improved most with the metronome beat (slope *b* = 0.86, *p* = 0.03), even though it also increased wrist flexor activity (*b* = 0.0021, *p* < 0.0001) and co-activation (*b* = 0.07, *p* = 0.004). Self-selected music did not increase wrist extension, but marginally increased flexor muscle activity (*b* = 0.0010, *p* = 0.04). Thus rhythmic auditory constraints improved motor control in subjects with spastic paresis who were at an earlier stage in motor recovery post-stroke. In the spastic co-contraction group (Figure 6B), wrist extension improved most *without* any auditory cueing (*b* = 1.83, *p* < 0.0001), which increased wrist extensor muscle activation (*b* = 0.004, *p* = 0.0002) and decreased co-activation across the wrist joint (*b* = −0.1, *p* = 0.0006). In contrast, self-selected music increased co-activation (*b* = 0.059, *p* = 0.04) in this group. Thus practice without auditory constraints was most beneficial in subjects with spastic co-contraction. In the minimal paresis group (Figure 6C), there was no improvement in wrist extension across the auditory conditions. The slope for wrist extension was most negative with happy sounds (*b* = −0.86, *p* = 0.03), wrist extensor activation decreased with the metronome beat (*b* = −0.0022, *p* = 0.02), and wrist flexor activation increased without auditory stimulation (*b* = 0.0012, *p* = 0.015).

## Discussion

Neurological and behavioral differences between patients and within each patient over the course of post-stroke recovery can influence how learning occurs during task-specific interactions. Hence, it is necessary to reconcile the vast clinical and movement heterogeneity in the post-stroke population to develop evidence-based rehabilitation protocols directed toward more homogenous groups of patients. Toward this end, the purpose of this study was to: (1) stratify subjects with post-stroke hemiparesis according to their stage of recovery using task-based kinematic measures, and (2) to examine how various types of auditory constraints interact with stage of recovery to facilitate learning of a wrist extension task with the paretic limb. The subjects were stratified into three distinct groups based on their speed and extent of wrist extension. Differences in clinical metrics and task performance led to the characterization of stage of recovery into three groups: (1) the spastic paresis group showed weak extensor drive with flexor spasticity and moderate co-activation of the flexors and extensors, and higher level of motor impairment; (2) the spastic co-contraction group showed higher flexor activation relative to the extensor and excessive co-activation of the flexors and extensors, with moderate level of motor impairment; and (3) the minimal paresis group showed restored extensor drive, low levels of co-activation, and minimal level of motor impairment. The effect of auditory constraints on rate of learning with the paretic hand after bimanual training was measured by the slope of wrist extension, and wrist extensor and flexor muscle activation patterns. Auditory stimulation with a metronome beat increased the rate of learning of wrist extension in subjects with spastic paresis, even though it increased flexor activation and co-activation across the flexor and extensor muscles. In contrast, bimanual training *without* auditory stimulation produced the greatest improvement in subjects with spastic co-contraction, increased wrist extensor activation, and reduced co-activation. Auditory stimulation in subjects with minimal paresis did not improve wrist extension, but performance was sensitive to the effects of auditory stimulation in this group. These results suggest that altering auditory task constraints during the same task can have different and even opposite effects on motor performance and learning in individuals at different stages of recovery post-stroke. These results cannot be explained by differences in proprioceptive sensation, task difficulty, or fatigue across the groups or conditions. The results further our understanding of possible mechanisms underlying progression of recovery from one stage to the next after stroke.

### Stratification of subjects reflects temporal stages in post-stroke recovery

Subjects with stroke have traditionally been classified based on the time elapsed since their stroke into acute (0–3 months), subacute (3–6 months), and chronic (6 months onward) categories. Recovery has been found to be most rapid in the acute and subacute periods (49), but recently compiled evidence shows that it continues well into the chronic period (50), although the trajectory of recovery may be punctuated by “plateaus” or temporary arrests in recovery. All the subjects in our study were in the chronic phase and may be considered to have plateaued. In longitudinal studies, increases in Fugl-Meyer scores suggest progression toward recovery. The Fugl-Meyer Scale is based on the observation of sequential recovery of motor function by Twitchell and Brunnstrom (6, 9, 10). It is the most widely used quantitative measure of motor recovery post-stroke (51, 52), and the scores have been shown to correlate with the extent of corticospinal tract damage (53). Hence, one can consider subjects with lower Fugl-Meyer scores as being more impaired or at an earlier stage in the recovery process compared to those with higher scores. In this study, subjects in the spastic paresis group had the lowest average Fugl-Meyer scores (both total and for the wrist and hand), which progressively increased in the spastic co-contraction and minimal paresis groups.

Fugl-Meyer scores have also been used to stratify subjects into groups (54, 55), but the cut-offs have been variable. Furthermore, the Fugl-Meyer Scale was constructed on the assumptions that recovery proceeds in a proximal-to-distal fashion and from synergistic-to-isolated movements (8, 51); however, both these assumptions have been contested recently (56–58). To circumvent the shortcomings of the Fugl-Meyer Scale in stratifying subjects, we used task-based kinematic measures, that is, speed and extent of wrist extension during the task, as direct, objective, and reliable measures of movement ability to stratify subjects into groups. Note that wrist movement amplitudes recorded during the task were lower than those recorded with goniometry prior to the task as would be expected due to the repetitive nature of the task.

We found that the spastic paresis group showed higher speed and amplitude of movement than the spastic co-contraction group, even though the Fugl-Meyer scores were higher in the spastic co-contraction group. This may seem surprising and contradictory to the notion of a linear improvement in movement ability over the course of post-stroke recovery. However, Twitchell observed that spasticity or tone continues to increase and reaches a peak before it starts to decrease (6). In this study, we measured spasticity clinically using the Modified Ashworth Scale, and by the extent of co-activation across the flexors and extensors during the task. We found that the spastic co-contraction group had equally increased tone in both the flexors and extensors, and 10 times greater co-activation on the paretic side than on the non-paretic side. While some degree of co-activation between the agonist and antagonist muscles is normal during movement, excessive co-activation leads to reduced movement speed and amplitude (59). Therefore, it follows that a progression of recovery from spastic paresis would lead to a dip in movement ability due to increases in co-activation before it begins to improve again as seen across our three groups. Our results suggest that the processes underlying progression of recovery are non-linear, and predict that movement kinematics and muscle activation patterns may worsen as recovery progresses and then get better. These predictions should be confirmed by future longitudinal studies that measure kinematics and EMG over time.

Furthermore, our results show that auditory constraints increase movement amplitude but also increase muscle co-activation in subjects with spastic paresis, suggesting that individuals at earlier stages of motor recovery benefit from an excitatory drive. In contrast, in subjects with spastic co-contraction, who were at a later stage in recovery and showed excessive co-activation from excitatory overdrive, auditory constraints were not helpful. Instead, bimanual-to-unimanual training *without* auditory stimulation led to reduced muscle co-activation and increased agonist muscle activity, suggesting that an inhibitory drive may be more beneficial to transition from spastic co-contraction. These findings are discussed further in the sections below. Thus, we propose that stratification of subjects based on relatively simple kinematic parameters of speed and extent of movement into the groups: (1) spastic paresis, (2) spastic co-contraction, and (3) minimal paresis reflects temporal stages in the course of post-stroke recovery, and transition from each of these stages may be triggered by specific constraints imposed during training.

### Rhythmic auditory stimulation improves performance in individuals with spastic paresis

At baseline, subjects with spastic paresis had both weakness and spasticity, defined as velocity-dependent increase in muscle tone at rest (60), as measured by the Modified Ashworth Scale (61). The emergence of spasticity is thought to reflect re-organization of the descending brainstem pathways leading to diffuse and synergistic patterns of movement. Weakness predominates in the early stages of spasticity (62), hence, while subjects in the spastic paresis group could activate their wrist extensor muscle, their range of wrist extension was limited. Spasticity was greater in the flexor muscles, consistent with the emergence of a flexor synergy pattern (10). Co-activation across the flexors and extensors was increased, but not disabling, as it did not hinder wrist extension (63, 64). In this group, auditory stimulation with a metronome beat in conjunction with bimanual training led to increased wrist extension, while that with self-selected music and happy sounds did not. However, both the metronome beat and self-selected music increased wrist flexor activation.

Both the metronome beat and self-selected music have rhythmic components; the rhythm was even and constant with a metronome, but uneven and changing with music. Both even and uneven rhythmic stimulation have been shown to increase muscle co-activation (65). The underlying mechanism is thought to be increased excitability of spinal motor neurons via the reticulospinal pathway, with facilitation of the H-reflex response (66, 67). Using functional MRI and effective connectivity analyzes, it has been shown that listening to music relative to scrambled musical sounds, activates a bilateral network of mesolimbic structures including the nucleus accumbens and the ventral tegmental area (38) leading to dopamine release and arousal. The ventral tegmental area in turn forms part of the midbrain reticular formation where the reticulospinal tracts originate. Excitation of the reticular formation is known to increase spasticity via the reticulospinal projections to the spinal cord (68). Thus, both the metronome beat and stimulating music can increase muscle tone and co-activation that may be helpful in earlier stages of recovery from flaccid paralysis. Non-musical and non-rhythmic auditory stimulation, as in our happy sounds condition, does not produce this effect. Furthermore the type of music, whether stimulating or relaxing, can modulate the extent of arousal and may produce a different effect on muscle tone.

However, only auditory stimulation with a metronome beat in conjunction with bimanual training led to increased unimanual wrist extension, while that with self-selected music and happy sounds did not. Even rhythms have been shown to reduce the variability in EMG responses, whereas uneven rhythms increase the variability in healthy individuals (65). Patients with stroke show disordered motor unit recruitment on EMG (69–72), but training to even metronome beats has been shown to decrease EMG variability (73) and improve motor outcomes post-stroke (23, 25, 27, 73–75). More efficient motor unit recruitment and sensorimotor synchronization (28) to the even metronome beat can explain the increased wrist extension without a notable increase in extensor activation as seen in our subjects with spastic paresis. In contrast, the variable rhythms in music and subtle differences in the type of music chosen, the tempo of the song and its match to the individual’s physical abilities may have influenced attention to the rhythmic component of music leading to a reduced peripheral synchronizing effect on wrist extension.

In healthy individuals, sensorimotor coupling to temporally structured auditory input has been shown to recruit a striato-thalamo-cortical-system involving basal ganglia, thalamus, premotor cortex (PMC), supplementary motor area (SMA), and dorsolateral prefrontal cortex [see Ref. (76) for review]. Simultaneous bimanual rhythmic movements involve interhemispheric coupling primarily in the PMC, posterior parietal cortex, and cerebellum (77), and switching from simultaneous bimanual synchronized movements to unimanual movements leads to a higher degree of interhemispheric connectivity involving the PMC, SMA, and sensorimotor areas (78). Furthermore, studying acallosal patients has shown that temporal coupling during rhythmic movements arises in large part from interactions between the two hemispheres (79). Taken together with these data, our results suggest that bimanual-to-unimanual movements synchronized to rhythmic auditory stimulation excites a bilateral distributed sensorimotor network, which may facilitate the progression of motor recovery in individuals with spastic paresis.

### Auditory stimulation does not improve inhibitory control in individuals with spastic co-contraction

When the threshold for reflex activity continues to reduce due to progressive re-organization of the supraspinal descending drive to the spinal cord, peripheral structures of the muscle, muscle spindles, and fascia are further shortened and spasticity evolves into stretch-sensitive forms such as spastic co-contraction (63). Spastic co-contraction refers to inappropriate antagonist recruitment triggered by volitional command (64). Clinically, spastic co-contraction opposes voluntary movement and contributes to impairment in active function, which was seen clearly in our subjects in this group where attempted wrist extension produced paradoxical wrist flexion. While some degree of co-activation between the agonist and antagonist muscles is normal during movement and necessary for joint stability, better movement accuracy and energy efficiency during functional activities, it has been shown to decrease with skill training (80–82). However, its persistence post-stroke signals disrupted reciprocal inhibition of antagonist muscles (83). Sensory feedback from muscle afferents mediates reciprocal inhibition through both spinal and cortical mechanisms (84, 85). Cortical suppression of the antagonist muscle is initiated centrally during preparation of agonist muscle contraction (86, 87) and the degree of suppression is proportional to the amplitude of stretch of the muscle (88).

Bilateral synchronous mirror symmetric flexion-extension movements have been shown to modulate cortical inhibition in neurologically intact individuals (89) and subjects with stroke (30). Somatosensory and visual information from each side of the body is processed bilaterally (90–92), and interlimb coordination is mediated by motor representations in the parietal and premotor areas shared by both limbs (93). Transcallosal pathways between homotopic regions of the two hemispheres (94–96) may also facilitate transmission of accurate sensory information from the intact hemisphere (33). Passive wrist extension on the affected side (which was facilitated by linked movements with the unaffected hand in this study) in severely impaired patients has been shown to produce fMRI changes in contralesional secondary sensorimotor areas in the ventral premotor and parietal cortices (97), which play a crucial role in re-organization of motor output. Thus, in patients with spastic co-contraction, bimanual training *without* auditory stimulation may restore sensory feedback, and reinstate reciprocal control in the paretic hand, aiding progression to the next stage of post-stroke recovery. In contrast, self-selected music may have continued to potentiate the stretch reflex through facilitation of descending spinal pathways in this group as discussed above.

### Individuals with minimal paresis show varied responses to auditory stimulation

In subjects with minimal paresis, there was little change in wrist extension across the auditory conditions perhaps due to a ceiling effect. Later stages of recovery have been shown to be mediated by re-organization in the ipsilesional cortex (16–18). Thus, it is not surprising that subjects in this group, who were farther along in their recovery, did not benefit substantially from either bimanual-to-unimanual training or auditory stimulation at the wrist. These strategies would perhaps still be applicable for training of hand and finger control. Subjects with minimal paresis no longer had significant spasticity or co-contraction, but were clearly still impaired compared to the unaffected side. The challenge in these subjects is fine-tuning of muscular control and restoration of dexterity, which may require different types of task constraint.

## Conclusion

This was a single-session study where bimanual-to-unimanual training of the paretic side was focused on improvement in performance and learning of a wrist extension task, as restoration of control at the wrist is especially challenging after stroke and necessary for hand function. The main purpose and novelty of this study is to show that auditory stimulation interacts with stage of recovery post-stroke to influence motor learning on a bimanual-to-unimanual wrist extension task. Several important conclusions may be drawn from this study. First, subjects in the chronic post-stroke period can be stratified based on simple movement kinematics to reflect their temporal stage of recovery, which may not be reflected by the time since stroke, and which in turn can inform the selection of strategies to drive subsequent progression of recovery post-stroke. Our data predict that during natural progression of post-stroke recovery, there could be a dip in movement ability due to increased co-contraction and then an increase in movement ability when co-contraction is inhibited. Second, our results show how different auditory constraints influence motor performance at various stages of recovery, perhaps through excitation and inhibition of distinct neural substrates. The effects of auditory constraints on muscle activation patterns provide insight into the mechanisms of transition across impairment levels, contributing to the understanding of how re-organization of CNS pathways may occur. Third, bimanual-to-unimanual learning can be a useful model to probe the rate of learning during single-session studies, providing an alternative to or a stratification tool prior to lengthy and expensive randomized control trials. We have recently found that long-term training locks-in the transient improvement seen during single-session bimanual-to-unimanual training (Preeti Raghavan, unpublished data). Together, the results lay the foundation for personalized protocols for post-stroke rehabilitation to advance the progression of recovery from one stage to the next, and hold significant implications for further research and clinical practice. Future work may confirm the effect of auditory constraints seen in our study on longitudinal progression of motor recovery in patients at different stages of recovery.

## Conflict of Interest Statement

The authors declare that the research was conducted in the absence of any commercial or financial relationships that could be construed as a potential conflict of interest.
